# Variational Bayesian-Based Improved Maximum Mixture Correntropy Kalman Filter for Non-Gaussian Noise

**DOI:** 10.3390/e24010117

**Published:** 2022-01-12

**Authors:** Xuyou Li, Yanda Guo, Qingwen Meng

**Affiliations:** Department of Intelligent Systems Science and Engineering, Harbin Engineering University, Harbin 150001, China; lixuyou@hrbeu.edu.cn (X.L.); mengqingwen@hrbeu.edu.cn (Q.M.)

**Keywords:** Kalman filter, maximum correntropy criterion, mixture correntropy, variational Bayesian inference

## Abstract

The maximum correntropy Kalman filter (MCKF) is an effective algorithm that was proposed to solve the non-Gaussian filtering problem for linear systems. Compared with the original Kalman filter (KF), the MCKF is a sub-optimal filter with Gaussian correntropy objective function, which has been demonstrated to have excellent robustness to non-Gaussian noise. However, the performance of MCKF is affected by its kernel bandwidth parameter, and a constant kernel bandwidth may lead to severe accuracy degradation in non-stationary noises. In order to solve this problem, the mixture correntropy method is further explored in this work, and an improved maximum mixture correntropy KF (IMMCKF) is proposed. By derivation, the random variables that obey Beta-Bernoulli distribution are taken as intermediate parameters, and a new hierarchical Gaussian state-space model was established. Finally, the unknown mixing probability and state estimation vector at each moment are inferred via a variational Bayesian approach, which provides an effective solution to improve the applicability of MCKFs in non-stationary noises. Performance evaluations demonstrate that the proposed filter significantly improves the existing MCKFs in non-stationary noises.

## 1. Introduction

The state estimation problem in dynamic systems is an important research topic in engineering applications and scientific research. As an excellent optimal state-space estimator, the Kalman filter (KF) is commonly applied in various fields like control systems and signal processing. Unfortunately, the optimality of KF requires exact system models and ideal noise conditions as summarized in [1].

The widely used KF, which usually refers to the Kalman filter based on the Hidden Markov Models (HMM), has rigorous requirements for the noise models. Both process and measurement noise are assumed as ideal independent Gaussian noise sequences. However, in practical applications, ideal noise conditions are not likely, and model uncertainties such as system structure changes and environmental disturbances are generally inevitable. Moreover, unexpected noise interference, such as colored noise and non-Gaussian noise, widely exist, and the performance of the KF is likely to worsen when applied to such situations. For example, when the independent noise assumption no longer holds, and the colored noise needs to be considered in the system model, as one of the efficient solutions, the pairwise Markov models (PMM), which can be deemed a general form of HMM, can be taken as an efficient improvement scheme for KF. In [2], the framework of the KF based on PMM is derived, which allows the cross-dependence between observations conditionally on hidden variables. As an extension, the KF based on triplet Markov chains is provided in [3]. These methods provided efficient solutions for the colored noise filtering from the perspective of the state-space models. On this basis, to solve the model parameters’ uncertain problems in practical applications. The robust parameter estimation problem for the PMM model is further explored in [4,5], which provided several representative schemes for the state estimation problems in colored noise or model parameter uncertain conditions.

On the other hand, the research on filtering algorithms under non-Gaussian noise has also attracted extensive attention. Generally, non-Gaussian distribution noises are often caused by impulsive noise, which gives the overall noise distributions obvious heavy-tailed features (such as some special Gaussian-mixture distributions). Extensive research has been carried out regarding the filtering problems in non-Gaussian noises. H-Infinity filtering and m-estimators are typical robust methods used for this purpose [6]. The Huber Kalman filter (HKF) is one of the most representative m-estimators, which retains a consistent form with KF and provides reliable robustness to external impulsive noise interference. In the existing relevant studies, various improvements based on the HKF have also been proposed, such as a batch mode [7] and several typical nonlinear schemes [8,9]. Besides m-estimators, student-t distribution filters have also been designed to deal with state estimate problems under heavy-tailed noise conditions. The filters attenuate the interference of outliers by their heavy-tailed distribution statistical characteristics [10,11]. However, student-t filters suffer from high-order statistics’ loss due to moment matching and inaccurate parameter selection, such as the degrees of freedom limiting their applicability. To solve the problem, another novel student-t filter based on the hierarchical Gaussian state-space model and variational Bayes (VB) inference approach is proposed, which is compactly called the robust student-t Kalman filter (RSTKF) [12]. Compared with the student-t filter, several unknown parameters, such as the degrees of freedom of the student-t distribution and the scale coefficient, are updated accordingly in the RSTKF [13,14,15].

Recently, a new robust maximum correntropy Kalman filter (MCKF) was proposed for the filtering problem in non-Gaussian noises [16,17]. The MCKF is derived on the maximum correntropy criterion (MCC), which introduces the Gaussian correntropy as a novel local similarity measure, and a novel object function based on correntropy is utilized to overcome the non-Gaussian noise interference [18]. Compared with other existing robust filters, the Gaussian correntropy-based MCKF has better statistical characteristics [19]. Meanwhile, the MCKF can be formulated in the same form as the KF. Similarly, the states constrained [20] and nonlinear forms [21,22] have been proposed and validated in various applications [23]. Moreover, an adaptive MCKF was proposed to improve the filtering performance by updating the measurement noise covariance via VB approach [24].

Due to the excellent robustness to outliers, in many cases, the filtering problem of a linear system corrupted by impulsive noise can be solved effectively by the MCKF. However, just like other robust filters, the filtering performance of the MCKF is closely related to its initial parameters, which are usually obtained by experience or trial and error method. Similar to many existing robust filtering algorithms with fixed parameters, it might result in performance degradation in non-stationary noises. Several studies have focused on the parameter problem of correntropy function. In prior works, heuristic solutions were proposed to adjust the kernel bandwidth during filtering [25,26]. As it is difficult to directly find an optimal kernel bandwidth during filtering, the mixture correntropy concept was proposed in [27,28], the method takes mixture Gaussian correntropy with different kernel parameters instead of the Gaussian correntropy, and a new maximum mixture correntropy criterion (MMCC) is derived from replacing MCC. However, the mixing probability of the mixture correntropy needs to be configured manually and fixed, which results in performance degradation similar to that of the MCKF.

To improve the filtering performance when applied to non-stationary noise conditions, it may be an efficient improved solution that considers the model’s switching scenarios. For instance, in [29], the optimal recursive filtering method is studied for non-Gaussian Markov switching models, in which a semi-supervised parameter estimation method is used. In addition, for the non-stationary noise conditions concerned in this paper, the variable Bayesian approach is an implementation scheme worthy of consideration. Based on the triplet Markov chain model, the general form of variable Bayesian inference is deduced in [30], and a structured variable Bayesian inference frame with regime switching is obtained. For typical linear filtering applications, the RSTKF proposed in [12] also adopts a similar operation scheme, where VB approximation is operated by rescaling the covariance and inferring parameters to deal with the non-stationary non-Gaussian noises. In [31], a similar problem is further studied, and a VB-based robust student’s t KF is applied to the linear PMM systems, which extends the filter’s applicability to more general conditions, and independent noise assumption is no longer needed. Therefore, inspired by the related references, the model switch concept is adopted in this work, and the variational Bayesian approach is also operated to infer estimation results. In view of the filtering accuracy degradation problem of MCKF in the non-stationary and non-Gaussian noise, a series of studies are carried out.

In this work, an improved maximum mixture Kalman filter (IMMCKF) is therefore proposed, the intermediate random variables are used to represent the mixing probability of mixture correntropy. The state variables and mixing probability are approximated by the derived variational Bayesian approach. Compared with the existing MCKF algorithms, the numerical test results demonstrate that the proposed filter could deal with the filtering problem well in a non-stationary non-Gaussian noises environment. The contributions of this paper are listed as follows:

(a) The accuracy degradation problem of existing fixed-parameter robust filtering algorithms in non-stationary noise environment is considered. By analyzing, it is inferred that the mixture correntropy function can be improved as the breakthrough point and applied to the filtering problem in such noise conditions. On this basis, a novel improved robust filtering algorithm is then further derived.
(b) By employing Beta-Bernoulli distributed intermediate random variables, a new hierarchical Gaussian state-space model is therefore derived, the system state vector, and the unknown variables are simultaneously estimated by utilizing the variational Bayesian technique.
(c) Through analyses and derivations, the necessary selection strategy of initial parameters is derived, and the numerical test results show that the filtering performance is enhanced obviously after several iterations. On the one hand, it achieved desired robust filtering performance when applied to non-stationary noise conditions. On the other hand, the proposed algorithm effectively avoids the possible filtering divergence difficulties of MCKF in practical application.

This paper is organized as follows: In Section 2, we review the concept of correntropy and existing MCKF. In Section 3, a variational Bayesian-based improved maximum mixture correntropy KF is derived to solve the filtering problem in non-stationary non-Gaussian noises, in which the variational Bayesian approach is applied in the proposed filter. Section 4 provides performance evaluations and analysis, demonstrating the advantages of the proposed filter in different noise conditions. Conclusions are given in Section 5.

## 2. Materials and Methods

### 2.1. Definition of Correntropy and Properties

Correntropy is a useful local similarity measure tool for state estimation in heavy-tailed noise environments. Given two random variables X,Y∈R with a joint distribution function $F_{XY}\left(x,y\right)$, correntropy can be defined as

(1) $$V\left(X,Y\right)=E\left[\kappa_{\sigma }\left(X,Y\right)\right]=\int \kappa_{\sigma }\left(x,y\right)dF_{XY}\left(x,y\right)$$

where $\kappa_{\sigma }\left(\cdot \right)$ is a shift-invariant Mercer kernel. In this work, the Gaussian kernel function is given by

(2) $$\kappa_{\sigma }\left(X,Y\right)\;=\;G_{\sigma }\left(e\right)\;=\;\exp\left(-\frac{e^{2}}{2\sigma^{2}}\right)$$

where e=X−Y, and σ>0 denotes the kernel bandwidth. In order to make correntropy applicable to complex noise conditions, the default Gaussian kernel function can be extended into a mixture correntropy form as follows:

(3) $$M\left(X,Y\right)=E[\rho G_{\sigma_{1}}\left(e\right)+\left(1-\rho \right)G_{\sigma_{2}}\left(e\right)]$$

where $G_{\sigma_{1}},G_{\sigma_{2}}$ represent the Gaussian correntropy with two different kernel bandwidth parameters, and ρ∈[0,1] represents the mixing probability. For convenience of application, (3) can be approximately expressed as

(4) $$M\left(X,Y\right)=\frac{1}{N}\left(\sum_{i=1}^{N}\rho \exp(-\frac{e_{i}^{2}}{2\sigma_{1}^{2}})+\left(1-\rho \right)\exp>(-\frac{e_{i}^{2}}{2\sigma_{2}^{2}})\right)$$

where *N* represents the number of samples. The mixture correntropy can be taken as a generalization of the original correntropy, if ρ=1 or ρ=0, it reduces to the Gaussian correntropy with single kernel parameter and M(X,Y)=1 if X=Y.

### 2.2. Robust Kalman Filter Based on Maximum Correntropy Criterion

Consider the linear state-space system based on HMM, the equations to be

(5) $$\begin{array}{cc} \; & x_{k}=F_{k}x_{k-1}+w_{k} \end{array}$$

(6) $$\begin{array}{cc} \; & y_{k}=H_{k}x_{k}+v_{k} \end{array}$$

where *k* is a discrete time index, $x_{k}\in R^{n}$ is the system state vector at discrete-time index *k*, $F_{k}$ is the state transition matrix, $y_{k}\in R^{m}$ is the measurement vector, $H_{k}$ is the measurement matrix; $w_{k}$ and $v_{k}$ are zero-mean process and measurement noise vector with nominal covariance $Q_{k}$ and $R_{k}$, respectively. It is assumed that both process and measurement noise are statistically independent and time uncorrelated. When the process and measurement noises are assumed ideal Gaussian distributions, and initial state $x_{0}$ is random Gaussian variables, the state can be inferred by the KF, which is an optimal filter in the minimum mean square error (MMSE) criterion. Additionally, the quadratic objective function can be formulated as follows:

(7) $${\hat{x}}_{k|k}=\arg\min_{x_{k}}J\left(x_{k}\right)=\arg\min_{x_{k}}\left(\frac{1}{2}{∥x_{k}-{\hat{x}}_{k|k-1}∥}_{P_{k|k-1}^{-1}}^{2}+\frac{1}{2}{∥y_{k}-H_{k}x_{k}∥}_{R_{k}^{-1}}^{2}\right)$$

where ${∥x∥}_{A}^{2}=x^{T}\mathrm{Ax}$. To minimize Equation (7), the KF is implemented in two steps as below, the prior estimation is

(8) $$\begin{array}{cc} \; & {\hat{x}}_{k|k-1}=F_{k}{\hat{x}}_{k-1|k-1} \end{array}$$

(9) $$\begin{array}{cc} \; & P_{k|k-1}=F_{k}P_{k-1|k-1}F_{k}^{T}+Q_{k-1} \end{array}$$

and the posterior measurement update is

(10) $$\begin{array}{cc} \; & K_{k}=P_{k|k-1}H_{k}^{T}{\left(H_{k}P_{k|k-1}H_{k}^{T}+R_{k}\right)}^{-1} \end{array}$$

(11) $$\begin{array}{cc} \; & {\hat{x}}_{k}={\hat{x}}_{k|k-1}+K_{k}\left(y_{k}-H_{k}{\hat{x}}_{k|k-1}\right) \end{array}$$

(12) $$\begin{array}{cc} \; & P_{k|k}=\left(I_{n}-K_{k}H_{k}\right)P_{k|k-1} \end{array}$$

where $P_{k|k-1}$ and $P_{k|k}$ represents the prior and posterior covariance matrix, respectively. The KF is an optimal estimator in an ideal white Gaussian noise environment. In Equation (7), only the second-order statistics are used during the state update, and the KF is susceptible to non-Gaussian noise interference.

To solve the filtering problem in non-Gaussian noise conditions, recently, MCC is often considered an efficient potential solution. As described in Section 2, correntropy has several necessary properties that make it capable of dealing with the non-Gaussian estimation problem. Different from the global similarity measure-mean square error (MSE), which only contains the second-order statistics, Gaussian correntropy incorporates all even order moments [17,18]. In geometric meaning, MSE gives the L2 norm distance while correntropy offers a hybrid norm distance, where the correntropy behaves like the L2→L0 norm with the increasing difference between two points.

As proved by the research in [17], maximizing the correntropy of two different random variables can be used as a criterion in dealing with the non-Gaussian noise problem, especially the filtering problem in heavy-tailed noises, which leads to MCC. Therefore, to enhance the robustness of the Kalman filter, the objective function based on MCC is introduced to replace the original quadratic cost function, and the new objective function therefore can be formulated as:

(13) $${\hat{x}}_{k}=\underset{x_{k}}{\mathrm{argmax}}J_{\mathrm{MCC}}\left(x_{k}\right)=\underset{x_{k}}{\arg\max}\left(\overset{n}{\underset{i=1}{\Sigma }}a_{i}G_{\sigma_{m}}\left(e_{i}\right)+\overset{m}{\underset{j=1}{\Sigma }}b_{j}G_{\sigma_{p}}\left(f_{j}\right)\right)$$

where $e={∥y_{k}-H_{k}{\hat{x}}_{k|k-1}∥}_{R_{k}^{-1}},f={∥{\hat{x}}_{k}-F_{k}{\hat{x}}_{k-1|k-1}∥}_{P_{k|k-1}^{-1}}$ the subscript *i* represents the *i*th element of the vector; a and b are tuning parameter vectors; and $\sigma_{m},\sigma_{p}$ denotes the kernel bandwidths corresponding to the $R_{k}$ and $P_{k|k-1}$, respectively. For simplicity, it is assumed that $\sigma_{m}=\sigma_{p}=\sigma$, to ensure the filter converges to the KF when the kernel bandwidth goes to infinity, assuming that $a_{i}\;=\;\sigma$, $b_{j}\;=\;\sigma$[21]. The prior estimation steps of the MCKF are the same as in Equations (8) and (9) and the posterior state estimation of $x_{k}$ is obtained by below KF like equations with the updated filter gain as

(14) $$\begin{array}{cc} \; & {\bar{K}}_{k}={\bar{P}}_{k|k-1}H_{k}^{T}{\left(H_{k}{\bar{P}}_{k|k-1}H_{k}^{T}+{\bar{R}}_{k}\right)}^{-1} \end{array}$$

(15) $$\begin{array}{cc} \; & {\bar{P}}_{k|k-1}=S_{P_{k|k-1}}\Lambda_{p}^{-1}S_{P_{k|k-1}}^{T} \end{array}$$

(16) $$\begin{array}{cc} \; & {\bar{R}}_{k}=S_{R_{k}}\Lambda_{m}^{-1}S_{R_{k}}^{T} \end{array}$$

where $\Lambda_{p}=\mathrm{diag}\left(G_{\sigma }\left(f_{1}\right)G_{\sigma }\left(f_{2}\right)...G_{\sigma }\left(f_{n}\right)\right)$, $\Lambda_{m}=\mathrm{diag}\left(G_{\sigma }\left(e_{1}\right)G_{\sigma }\left(e_{2}\right)...G_{\sigma }\left(e_{m}\right)\right)$, and $P_{k|k-1}=S_{P_{k|k-1}}S_{P_{k|k-1}}^{T}$, $R_{k}=S_{R_{k}}S_{R_{k}}^{T}$. It is worth noting that the MCC solution cannot be obtained in a closed form and usually solves it using an iterative update algorithm such as the fixed-point iterative algorithm, which involves no step size and may converge fast, the condition that guarantees the convergence of the fixed point MCC was given in [32].

## 3. Main Results

### 3.1. Robust Kalman Filter Based on Mixture Correntropy Criterion

The kernel parameters of Gaussian correntropy determine the filtering performance of the MCKF, and an improper kernel bandwidth might lead to filtering performance degradation or even diverge. For this reason, the mixture correntropy is an alternative solution to improve the solution of problem by reducing the filter’s sensitivity to the kernel parameters.

The maximum mixture correntropy Kalman filter (MMCKF) is derived in this section. According to the mixture correntropy Function (4), the objective function of the maximum mixture correntropy criterion can be formulated as

(17) $${\hat{x}}_{k}=\underset{x_{k}}{\arg\max}J_{\mathrm{MMCC}}\left(x_{k}\right)=\underset{x_{k}}{\arg\max}\left(\overset{n}{\underset{i=1}{\Sigma }}a_{i}M\left(e_{i}\right)+\overset{m}{\underset{j=1}{\Sigma }}b_{j}M\left(f_{j}\right)\right)$$

where $M(e_{i})$ represents the mixture correntropy function as (3). Similar to Equation (13), it is assumed that $a_{i}=b_{j}=\lambda$, and mixture correntropy is the convex combination of the two Gaussian correntropy functions. To maximize this objective function, the solution can be obtained by solving

(18) $$\frac{\partial J_{\mathrm{MMCC}}\left(x_{k}\right)}{\partial x_{k}}=\lambda \left(\overset{n}{\underset{i=1}{\Sigma }}{Ø}_{i}M\left(e_{i}\right)+\overset{m}{\underset{j=1}{\Sigma }}{Ø}_{j}M\left(f_{j}\right)\right)=0$$

where

(19) $${Ø}_{i}=\left(\frac{\rho G_{\sigma_{1}}\left(e_{i}\right)}{\sigma_{1}^{2}}+\frac{\left(1-\rho \right)G_{\sigma_{2}}\left(e_{i}\right)}{\sigma_{2}^{2}}\right)$$

represents the derivation of the correntropy function.

To maintain consistency to the KF, a tuning factor λ should be properly assigned as

(20) $$\lambda =\frac{\sigma_{1}^{2}\sigma_{2}^{2}}{\rho \sigma_{2}^{2}+\left(1-\rho \right)\sigma_{1}^{2}}$$

and the MMCKF converges to the optimal KF while the process and measurement noises obey ideal Gaussian distributions. A modified mixture correntropy function $C(e_{i})$ is then formulated as

(21) $$C\left(e_{i}\right)=\lambda {Ø}_{i}=\mu G_{\sigma_{1}}\left(e_{i}\right)+\left(1-\mu \right)G_{\sigma_{2}}\left(e_{i}\right)$$

with μ=ρσ22ρσ22(ρσ22+(1−ρ)σ12)(ρσ22+(1−ρ)σ12), and the optimal estimate of $x_{k}$ is obtained by KF like equations, where $\Lambda_{p}=\mathrm{diag}\left(C\left(f_{1}\right)C\left(f_{2}\right)...C\left(f_{n}\right)\right)$, $\Lambda_{m}=\mathrm{diag}\left(C\left(e_{1}\right)C\left(e_{2}\right)...C\left(e_{m}\right)\right)$, and μ∈[0,1]. Here, $C(e_{i})$ can be regarded as a linear transformation of $M(e_{i})$, they are positive correlated, and $C\left(e_{i}\right)=M\left(e_{i}\right)$ if $\rho ,\mu \in \left\{0,1\right\}$.

### 3.2. Improved MMCKF via Variational Bayesian Approximation

Similar to the Gaussian correntropy of the MCKF, in Equation (21), the mixing probability parameters are constant, which inevitably results in the filtering performance degradation in non-stationary noises. To improve the filtering performance in such complex noise environments, an improved algorithm is derived in this section. The mixing probability of the mixture correntropy is reassigned as an unknown random variable that can be further approximated during filtering.

According to the Bayesian theorem, the posterior probability density function (PDF) $p(x_{k}|y_{1:k})$ is formulated as

(22) $$p\left(x_{k}|y_{1:k}\right)\propto p\left(y_{k}|x_{k}\right)p\left(x_{k}|y_{1:k-1}\right)$$

in which $p(x_{k}|y_{1:k-1})$ is determined using the Chapman–Kolmogorov equation. Therefore,

(23) $$p\left(x_{k}|y_{1:k-1}\right)\propto \int p\left(x_{k}|x_{k-1}\right)p\left(x_{k-1}|y_{1:k-1}\right)dx_{k-1}$$

where $p(x_{k}|x_{k-1})$ is the posterior density at time k−1 and $p(x_{k-1}|y_{1:k-1})$ is the transition density.

In this work, we assumed that μ is an unknown random variable, and μ∈[0,1]. In order to approximate the unknown state and reasonable mixing probability via VB approach, we first need to determine the assumed distribution of unknown variable. Then, it is necessary to introduce a prior probability distribution p(μ). According to the desired scenario, μ is assumed to be an Beta distributed variable. Since the likelihood distribution of the mixing probability μ can be formulated as a Bernoulli distribution by introducing Bernoulli random variables ξ, according to the Bayesian probability theory, we have

(24) $$p\left(\mu |\xi \right)=\frac{p(\xi |\mu )\cdot p(\mu )}{\int p(\xi |\mu )\cdot p(\mu )d\xi }$$

and then, p(μ|ξ)∝p(ξ|μ)·p(μ). According to the Bayesian formula, the posterior probability distribution is directly proportional to the product of the prior probability distribution and the likelihood function. The form of the posterior probability distribution will be the same as that of the prior. Therefore, the conjugate prior distribution of μ is selected as a Beta distribution according to Bayesian probability theory, have $p\left(\mu \right)=Be\left(\mu ;a,b\right)$, where E(μ)=a/(a+b).

Now that we have determined the distribution of the required intermediate variables, we can further derive the following specific formula based on the system model. Using two random variables $s_{k}$ and $t_{k}$ obey Bernoulli distribution to generate the likelihood distribution. In order to ensure that the prior and posterior probability distributions retain the same form, the Beta distribution is taken as the prior conjugation distribution for inference. Therefore, the conditional probability $p(t_{k}|\alpha_{k})$ and $p(s_{k}|\beta_{k})$ can be formulated as

(25) $$\begin{array}{cc} \; & p\left(t_{k}|\alpha_{k}\right)=\alpha_{k}^{t_{k}}{\left(1-\alpha_{k}\right)}^{\left(1-t_{k}\right)},s.t.t_{k}\in \left\{0.1\right\} \end{array}$$

(26) $$\begin{array}{cc} \; & p\left(s_{k}|\beta_{k}\right)=\beta_{k}^{s_{k}}{\left(1-\beta_{k}\right)}^{\left(1-s_{k}\right)},s.t.s_{k}\in \left\{0.1\right\} \end{array}$$

in which $\alpha_{k}$ and $\beta_{k}$ obey the Beta distribution as

(27) $$\begin{array}{cc} \; & p\left(\alpha_{k}\right)=\mathrm{Be}(\alpha_{k};a_{0},b_{0}) \end{array}$$

(28) $$\begin{array}{cc} \; & p\left(\beta_{k}\right)=\mathrm{Be}(\beta_{k};c_{0},d_{0}) \end{array}$$

where $a_{0},b_{0},c_{0}$ and $d_{0}$ represent the prior Beta parameters for the mixing probabilities. Then, the conditional PDF $p(y_{k}|x_{k},t_{k})$ and $p(x_{k}|y_{1:k-1},s_{k})$ can be rewritten as the following hierarchical Gaussian form by

(29) $$\begin{array}{cc} \; & p\left(y_{k}|x_{k},t_{k}\right)=N{\left(y_{k};H_{k}x_{k},{\tilde{R}}_{k}\right)}^{t_{k}}N{\left(y_{k};H_{k}x_{k},{\bar{R}}_{k}\right)}^{\left(1-t_{k}\right)} \end{array}$$

(30) $$\begin{array}{cc} \; & p\left(x_{k}|y_{1:k-1},s_{k}\right)=N{\left(x_{k};{\hat{x}}_{k|k-1},{\tilde{P}}_{k|k-1}\right)}^{s_{k}}N{\left(x_{k};{\hat{x}}_{k|k-1},{\bar{P}}_{k|k-1}\right)}^{\left(1-s_{k}\right)} \end{array}$$

where ${\tilde{R}}_{k}$, ${\tilde{P}}_{k|k-1}$ and ${\bar{R}}_{k}$, ${\bar{P}}_{k|k-1}$ represent the updated covariance matrix corresponding to the Gaussian correntropy functions $G_{\sigma_{1}}$ and $G_{\sigma_{2}}$, respectively. The modified prior error covariance is formulated as ${\tilde{P}}_{k|k-1}=S_{P_{k|k-1}}\Lambda_{p,\sigma_{1}}^{-1}S_{P_{k|k-1}}^{T}$ and ${\bar{P}}_{k|k-1}=S_{P_{k|k-1}}\Lambda_{p,\sigma_{2}}^{-1}S_{P_{k|k-1}}^{T}$, and the measurement noise covariance is formulated as ${\tilde{R}}_{k}=S_{R_{k}}\Lambda_{m,\sigma_{1}}^{-1}S_{R_{k}}^{T}$ and ${\bar{R}}_{k}=S_{R_{k}}\Lambda_{m,\sigma_{2}}^{-1}S_{R_{k}}^{T}$.

Therefore, the conditional probability density distribution of $p(y_{k}|x_{k},\alpha_{k},t_{k})$ and $p(x_{k}|y_{1:k-1},\beta_{k},s_{k})$ can be formulated as

(31) $$\begin{array}{cc} \; & p\left(y_{k}|x_{k},\alpha_{k},t_{k}\right)=N{\left(y_{k};H_{k}x_{k},{\tilde{R}}_{k}\right)}^{t_{k}}\\ \; & N{\left(y_{k};H_{k}x_{k},{\bar{R}}_{k}\right)}^{\left(1-t_{k}\right)}p\left(t_{k}|\alpha_{k}\right)p\left(\alpha_{k}\right)s.t.\;t_{k}\in \left\{0,1\right\} \end{array}$$

(32) $$\begin{array}{cc} \; & p\left(x_{k}|y_{1:k-1},\beta_{k},s_{k}\right)=N{\left(x_{k};{\hat{x}}_{k|k-1},{\tilde{P}}_{k|k-1}\right)}^{s_{k}}\\ \; & N{\left(x_{k};{\hat{x}}_{k|k-1},{\bar{P}}_{k|k-1}\right)}^{\left(1-s_{k}\right)}p\left(s_{k}|\beta_{k}\right)p\left(\beta_{k}\right)s.t.\;s_{k}\in \left\{0,1\right\} \end{array}$$

According to the likelihood PDF derived above and the Bayesian theorem, the joint PDF can be given as follows:

(33) $$\begin{array}{c} p\left(\Theta_{k},y_{1:k}\right)=p\left(y_{1:k-1}\right)N{\left(x_{k};{\hat{x}}_{k|k-1},{\tilde{P}}_{k|k-1}\right)}^{s_{k}}N{\left(x_{k};{\hat{x}}_{k|k-1},{\bar{P}}_{k|k-1}\right)}^{\left(1-s_{k}\right)}N{\left(y_{k};H_{k}x_{k},{\tilde{R}}_{k}\right)}^{t_{k}}\\ N{\left(y_{k};H_{k}x_{k},{\bar{R}}_{k}\right)}^{1-t_{k}}\alpha_{k}^{t_{k}}{\left(1-\alpha_{k}\right)}^{\left(1-t_{k}\right)}\beta_{k}^{s_{k}}{\left(1-\beta_{k}\right)}^{\left(1-s_{k}\right)}\mathrm{Be}\left(\alpha_{k};a_{0},b_{0}\right)\mathrm{Be}\left(\beta_{k};c_{0},d_{0}\right) \end{array}$$

where $\Theta_{k}\overset{\Delta }{=}\left\{x_{k},s_{k},t_{k},\alpha_{k},\beta_{k}\right\}$ contains the state variable $x_{k}$ that needs to be estimated, and the Beta-Bernoulli variables $\left\{\alpha_{k},\beta_{k}\right\},\left\{s_{k},t_{k}\right\}$ are used for the inference of mixing probability parameters. Therefore, to obtain the estimated value of Θ using the VB inference, according to prior work [12,33], the approximate posterior PDF of the element in Θ needs to satisfy the following equation:

(34) $$\log q\left(\theta \right)=E_{\Theta_{k}^{-\theta }}\left[\log p\left(\Theta_{k},y_{1:k}\right)\right]+C_{\theta }$$

where θ is an element of $\Theta_{k}$, $\Theta^{-\theta }$ means the remain elements of Θ except θ, and $C_{\theta }$ is a constant with respect to θ. As (34) cannot be solved analytically, fixed-point iteration is required to achieve an approximate solution. By expanding (34), the following can be obtained:

(35) $$\begin{array}{c} \begin{array}{c} \log p\left(\Theta_{k},y_{1:k}\right)=\left(1-s_{k}\right)\log N\left(x_{k};{\hat{x}}_{k|k-1},{\bar{P}}_{k|k-1}\right)+s_{k}\mathrm{logN}\left(x_{k};{\hat{x}}_{k|k-1},{\tilde{P}}_{k|k-1}\right)\\ +\left(1-t_{k}\right)\mathrm{logN}\left(y_{k};H_{k}x_{k},{\bar{R}}_{k}\right)+t_{k}\log N\left(y_{k};H_{k}x_{k},{\tilde{R}}_{k}\right)+t_{k}\log\alpha_{k}+\left(1-t_{k}\right)\log\left(1-\alpha_{k}\right) \end{array}\\ +s_{k}\log\beta_{k}+\left(1-s_{k}\right)\log\left(1-\beta_{k}\right)+\mathrm{logBe}\left(\alpha_{k};a_{0},b_{0}\right)+\log\mathrm{Be}\left(\beta_{k};c_{0},d_{0}\right)+C_{\Theta_{k}} \end{array}$$

Initialize the parameters $a_{0},b_{0},c_{0},d_{0}$ and calculate $E^{\left(i+1\right)}\left[t_{k}\right]=a_{0}/\left(a_{0}+b_{0}\right)$, $E^{\left(i+1\right)}\left[s_{k}\right]=c_{0}/\left(c_{0}+d_{0}\right)$, note that $E^{\left(0\right)}\left[\log\left(\alpha_{0}\right)\right]=\psi \left(a_{0}\right)-\psi \left(a_{0}+b_{0}\right)$, $E^{\left(0\right)}\left[\log\left(\beta_{0}\right)\right]=\psi \left(c_{0}\right)-\psi \left(c_{0}+d_{0}\right)$, where ψ represents the digamma function.

By exploiting (35), the estimation of the unknown parameters in $\Theta_{k}$ is implemented by fixed-point iteration loop in (a)–(c).

(a) Let $\theta =x_{k}$, utilizing (34) in (35), and $\log q^{\left(i+1\right)}\left(x_{k}\right)$ can be rewritten as

(36) $$\begin{array}{c} \begin{array}{c} \log q^{\left(i+1\right)}\left(x_{k}\right)=E^{\left(i\right)}\left[s_{k}\right]\log N\left(x_{k};H_{k}{\hat{x}}_{k|k}^{\left(i\right)},{\tilde{P}}_{k|k}^{\left(i\right)}\right)+\left(1-E^{\left(i\right)}\left[s_{k}\right]\right)\log N\left(x_{k};H_{k}{\hat{x}}_{k|k}^{\left(i\right)},{\bar{P}}_{k|k}^{\left(i\right)}\right) \end{array}\\ E^{\left(i\right)}\left[t_{k}\right]\log N\left(y_{k};H_{k}x_{k},{\tilde{R}}_{k}^{\left(i\right)}\right)+\left(1-E^{\left(i\right)}\left[t_{k}\right]\right)\log N\left(y_{k};H_{k}x_{k},{\bar{R}}_{k}^{\left(i\right)}\right)+C_{x_{k}} \end{array}$$

where *i* represents the *i*th fixed-point iterations. Using $E^{\left(i+1\right)}\left[s_{k}\right]$ and $E^{\left(i+1\right)}\left[t_{k}\right]$ replace the mixing probability ρ in (21) and then calculate the conditional PDF $p(x_{k}|y_{1:k-1})$ and $p(y_{k}|x_{k})$ by proposed mixture correntropy as

(37) $$q^{\left(i+1\right)}\left(x_{k}|y_{1:k-1}\right)q^{\left(i+1\right)}\left(y_{k}|x_{k}\right)=N\left(x_{k};{\hat{x}}_{k-1},{\hat{P}}_{k|k-1}\right)N\left(y_{k};H_{k}x_{k},{\hat{R}}_{k}\right)$$

where the modified prediction error covariance matrix ${\hat{P}}_{k|k-1}^{\left(i+1\right)}$ and the modified measurement noise covariance matrix ${\hat{R}}_{k}^{\left(i+1\right)}$ are used. After deformation, the posterior PDF $q^{\left(i+1\right)}\left(x_{k}\right)$ can be formulated as

(38) $$q^{\left(i+1\right)}\left(x_{k}\right)\propto N\left(x_{k};H_{k}{\hat{x}}_{k|k-1}^{\left(i\right)},{\hat{P}}_{k|k-1}^{\left(i\right)}\right)N\left(y_{k};H_{k}x_{k},{\hat{R}}_{k}^{\left(i\right)}\right)$$

According to (38), $q^{\left(i+1\right)}\left(x_{k}\right)$ is updated using a nominal Gaussian distribution as $q^{\left(i+1\right)}\left(x_{k}\right)=N\left(x_{k};H_{k}{\hat{x}}_{k|k}^{\left(i\right)},{\hat{P}}_{k|k}^{\left(i+1\right)}\right)$. Then ${\hat{x}}_{k|k}^{\left(i\right)}$ and the corresponding estimation error covariance matrix ${\hat{P}}_{k|k}^{\left(i+1\right)}$ are obtained by the measurement update of the KF as shown in (10)–(12) with ${\hat{P}}_{k|k-1}^{\left(i+1\right)}$ and ${\hat{R}}_{k}^{\left(i+1\right)}$.

(b) As for the expectation of the Bernoulli distribution, $E^{\left(i+1\right)}\left[s_{k}\right]$ and $E^{\left(i+1\right)}\left[t_{k}\right]$ can be updated as follows:

(39) $$\begin{array}{cc} \; & E^{\left(i+1\right)}\left[s_{k}\right]=\frac{{\mathrm{Pr}}^{\left(i+1\right)}\left(s_{k}=1\right)}{{\mathrm{Pr}}^{\left(i+1\right)}\left(s_{k}=1\right)+{\mathrm{Pr}}^{\left(i+1\right)}\left(s_{k}=0\right)} \end{array}$$

(40) $$\begin{array}{cc} \; & E^{\left(i+1\right)}\left[t_{k}\right]=\frac{{\mathrm{Pr}}^{\left(i+1\right)}\left(t_{k}=1\right)}{{\mathrm{Pr}}^{\left(i+1\right)}\left(t_{k}=1\right)+{\mathrm{Pr}}^{\left(i+1\right)}\left(t_{k}=0\right)} \end{array}$$

By setting $\theta =s_{k}$ and $\theta =t_{k}$, and then updating $q^{\left(i+1\right)}\left(s_{k}\right)$ and $q^{\left(i+1\right)}\left(t_{k}\right)$ using the Bernoulli distribution, the probabilities of $s_{k}$ and $t_{k}$ being 1 are given by:

(41) $$\begin{array}{cc} \mathrm{Pr}(t_{k}=1) & =\Delta_{1}^{i+1}\exp\left\{E^{\left(i\right)}\left[\log\alpha_{k}\right]+0.5\mathrm{tr}\left(\log\Lambda_{m,\sigma_{1}}\right)-0.5\mathrm{tr}\left(A_{k}^{i+1}{\bar{R}}_{k}^{-1}\right)\right\} \end{array}$$

(42) $$\begin{array}{cc} \mathrm{Pr}(s_{k}=1) & =\Delta_{2}^{i+1}\exp\left\{E^{\left(i\right)}\left[\log\beta_{k}\right]+0.5\mathrm{tr}\left(\log\Lambda_{p,\sigma_{1}}\right)-0.5\mathrm{tr}\left(B_{k}^{i+1}{\bar{P}}_{k|k-1}^{-1}\right)\right\} \end{array}$$

where Δ is a normalizing constant, and two auxiliary variables A,B are used to evaluate the prior probability covariance and measurement covariance as

(43) $$\begin{array}{cc} \; & A_{k}^{\left(i+1\right)}=E^{\left(i+1\right)}\left[\left(y_{k}-H_{k}x_{k}\right){\left(y_{k}-H_{k}x_{k}\right)}^{T}\right] \end{array}$$

(44) $$\begin{array}{cc} \; & B_{k}^{\left(i+1\right)}=E^{\left(i+1\right)}\left[\left(x_{k}-{\hat{x}}_{k|k-1}\right){\left(x_{k}-{\hat{x}}_{k|k-1}\right)}^{T}\right] \end{array}$$

To implement the fixed-point iteration, (43) and (44) is approximated as

(45) $$\begin{array}{cc} \; & \begin{array}{c} A_{k}^{\left(i+1\right)}=\left(y_{k}-H_{k}{\hat{x}}_{k|k}^{\left(i+1\right)}\right){\left(y_{k}-H_{k}{\hat{x}}_{k|k}^{\left(i+1\right)}\right)}^{T}+H_{k}P_{k|k}^{i+1}H_{k}^{T} \end{array} \end{array}$$

(46) $$\begin{array}{cc} \; & \begin{array}{c} B_{k}^{\left(i+1\right)}=P_{k|k}^{\left(i+1\right)}+\left({\hat{x}}_{k|k}^{\left(i+1\right)}-{\hat{x}}_{k|k-1}\right){\left({\hat{x}}_{k|k}^{\left(i+1\right)}-{\hat{x}}_{k|k-1}\right)}^{T} \end{array} \end{array}$$

(c) Next, $q^{\left(i+1\right)}\left(\alpha_{k}\right)$ and $q^{\left(i+1\right)}\left(\beta_{k}\right)$ are updated using the Beta distribution as shown in (27) and (28). Take the PDF of the Beta distribution into derivation, and, by setting $\theta =\alpha_{k}$ and $\theta =\beta_{k}$, then

(47) $$\begin{array}{cc} \; & q^{\left(i+1\right)}\left(\alpha_{k}\right)=\left(a_{0}-E^{\left(i+1\right)}\left[t_{k}\right]\right)\log\left(\alpha_{k}\right)+\left(b_{0}+E^{\left(i+1\right)}\left[t_{k}\right]-1\right)\log\left(1-\alpha_{k}\right)+C_{\alpha } \end{array}$$

(48) $$\begin{array}{cc} \; & q^{\left(i+1\right)}\left(\beta_{k}\right)=\left(c_{0}-E^{\left(i+1\right)}\left[s_{k}\right]\right)\log\left(\beta_{k}\right)+\left(d_{0}+E^{\left(i+1\right)}\left[s_{k}\right]-1\right)\log\left(1-\beta_{k}\right)+C_{\beta } \end{array}$$

where $q^{\left(i+1\right)}\left(\alpha_{k}\right)$ and $q^{\left(i+1\right)}\left(\beta_{k}\right)$ can be updated with new shape parameters, therefore, the shape parameters of the Beta prior distribution can be updated as follows:

(49) $$\begin{array}{cc} \; & a_{k}^{\left(i+1\right)}=a_{0}+E^{\left(i+1\right)}\left[t_{k}\right] \end{array}$$

(50) $$\begin{array}{cc} \; & b_{k}^{\left(i+1\right)}=b_{0}-E^{\left(i+1\right)}\left[t_{k}\right]+1 \end{array}$$

(51) $$\begin{array}{cc} \; & c_{k}^{\left(i+1\right)}=c_{0}+E^{\left(i+1\right)}\left[s_{k}\right] \end{array}$$

(52) $$\begin{array}{cc} \; & d_{k}^{\left(i+1\right)}=d_{0}-E^{\left(i+1\right)}\left[s_{k}\right]+1 \end{array}$$

After completing c), checking the convergence of the iteration according to the preset error threshold, if $\frac{∥{\hat{x}}_{k}^{\left(i+1\right)}-{\hat{x}}_{k}^{\left(i\right)}∥}{∥{\hat{x}}_{k}^{\left(i\right)}∥}<\varepsilon$ then stop the iteration and output the result. Otherwise, return to (a) for the next iteration.

In addition to the previous state and measurement vector, to begin the algorithm, the proposed filter only needs two kernel parameters and the initial prior beta shape parameters for the mixture of Gaussian correntropy. Among them, the kernel parameters of the Gaussian correntropy subfunction are more likely to be chosen by experience. In most cases, they have minimal impact on the filtering performance. This conclusion will be confirmed in the subsequent simulations.

On the other hand, the chosen of prior Beta distribution parameters needs a simple discussion. By combining the characteristics of the Beta distribution, the initial mixing probability ρ is determined by its prior shape parameters. If $a_{0}=c_{0}=0$ or $b_{0}=d_{0}=0$, the proposed filter converges to the original MCKF. In order to reduce input parameters and simplify the algorithm, it is generally assumed that $a_{0}=c_{0}$ and $b_{0}=d_{0}$ in most cases.

In this work, two Gaussian correntropies with different kernel parameters are used to cope with the stable filtering process and the process corrupted by dynamic abnormal errors, such as impulsive noise disturbance. In order to ensure filtering accuracy and stability, the prior parameters are not arbitrarily set, but obey a certain regularity. For example, in an ideal Gaussian noise environment, we have $E^{\left(j+1\right)}\left[t_{k}\right]\to 1$ and then the performance of the mixture correntropy converge to the Gaussian correntropy with a large kernel parameter, so that it has basic robustness and retains convergence to KF as much as possible, expanding (41) and we can find that

(53) $$\begin{array}{cc} E^{\left(i+1\right)}\left[t_{k}\right] & =\frac{p^{\left(i+1\right)}\left(t_{k}=1\right)}{p^{\left(i+1\right)}\left(t_{k}=1\right)+p^{\left(i+1\right)}\left(t_{k}=0\right)}\\ \; & =\frac{1}{1+p^{\left(i+1\right)}\left(t_{k}=0\right)/p^{\left(i+1\right)}\left(t_{k}=1\right)}\\ \; & =\frac{1}{1+\exp(E^{\left(i+1\right)}\left(1-\alpha_{0}\right)-E^{\left(i+1\right)}\left(\alpha_{0}\right)+C_{k})}\\ \; & =\frac{1}{1+\exp(\psi \left(b_{0}\right)-\psi \left(a_{0}\right)+C_{k})}\to 1 \end{array}$$

where $C_{k}$ represents the terms independent of $a_{0}$ and $b_{0}$. According to (41) and (42), if the distributions of measurement and state noise nearly meet the ideal Gaussian assumption is needed.

In contrast, for the filtering process corrupted by severe impulsive noise, the mixing probability is likely to be redistributed appropriately to enhance the robustness to abnormal noise, and then

(54) $$\begin{array}{cc} E^{\left(i+1\right)}\left[t_{k}\right] & =\frac{1}{1+p^{\left(i+1\right)}\left(t_{k}=0\right)/p^{\left(i+1\right)}\left(t_{k}=1\right)}\to 0\\ \; & \Rightarrow \exp(\psi \left(b_{0}\right)-\psi \left(a_{0}\right)+C_{k})>>0 \end{array}$$

As the definition of Gaussian correntropy Equation (2), if $\sigma_{1}>\sigma_{2}$, for the residual term $e_{i}$ with significant abnormality, there is a great of difference between $G_{\sigma_{1}}\left(e_{i}\right)$ and $G_{\sigma_{2}}\left(e_{i}\right)$, and $G_{\sigma_{1}}\left(e_{i}\right)\gg G_{\sigma_{2}}\left(e_{i}\right)$. Therefore, it is further inferred that $C_{k}$ increases significantly and then $\psi \left(b_{0}\right)-\psi \left(a_{0}\right)+C_{k}\gg 0$ at this time, where $\psi \left(b_{0}\right)-\psi \left(a_{0}\right)$ can be taken as a constant term to regulate the transition of $E^{\left(i+1\right)}\left[t_{k}\right]$, and $a_{0}>b_{0}$ still applies to this case.

Therefore, in this work, the parameters can be chosen within the range $0.95\leq a_{0}\leq 0.85$ and $b_{0}=1-a_{0}$, which can be used as a typical parameter configuration. On this basis, it may sometimes be useful to make a minor adjustment on the initial parameter configurations according to the specified application. This will contributes to further improving the algorithm’s performance in complex environments. The related results are shown in the later simulation.

## 4. Performance Evaluations and Analysis

### 4.1. Example I: 2-D Moving Target Tracking Model

Consider a two-dimensional (2D) moving target tracking system as

(55) $$F=\left[\begin{array}{cccc} 1 & \frac{\sin(wT)}{w} & 0 & -\frac{\left(1-\cos(wT)\right)}{w}\\ 0 & \cos(wT) & 0 & -\sin(wT)\\ 0 & -\frac{\left(1-\cos(wT)\right)}{w} & 1 & \frac{\sin(wT)}{w}\\ 0 & \sin(wT) & 0 & \cos(wT) \end{array}\right],\Gamma =\left[\begin{array}{cc} 0.5T^{2} & 0\\ T & 0\\ 0 & 0.5T^{2}\\ T & 0 \end{array}\right],H=\left[\begin{array}{cccc} 1 & 0 & 0 & 0\\ 0 & 0 & 1 & 0 \end{array}\right]$$

in which Γ represents the process noise gain matrix, T=0.2, w=0.2, and the total simulation steps N=200/T. It is assumed that $Q_{k}=0.1I_{2}$ and $R_{k}=10I_{2}$. The true initial state $x_{0}={\left[1\;1\;1\;1\right]}^{T}$, the initial state estimation ${\hat{x}}_{0}={\left[0\;0\;0\;0\right]}^{T}$ and the error covariance matrix $P_{0|0}=I_{4}$. In addition to the KF, the HKF with loss function parameter r = 1.345; the MCKF with typical kernel parameters σ = 2, σ = 3, σ = 5 and σ = 9, are used for comparison. For the algorithm proposed in this paper, we chose $\sigma_{1}$ = 9, $\sigma_{2}$ = 3 and $a_{0}=0.9$ as the default initial parameters. The maximum iteration times $N_{m}$ = 10 for the robust filters. The numerical test was coded with MATLAB and executed on a computer with Intel Core i7-9700 CPU @3.0 GHz.

In order to evaluate the filtering performance, the root mean square errors (RMSE) and averaged RMSE (ARMSE) are chosen as evaluation indicators of position and velocity estimation, which are defined as follows:

(56) $$\left\{\begin{array}{c} \mathrm{RMSE}(k)\overset{\Delta }{=}\sqrt{\frac{1}{M}\sum_{s=1}^{M}\left({\left(x_{k}^{s}-{\hat{x}}_{k}^{s}\right)}^{2}+{\left(y_{k}^{s}-{\hat{y}}_{k}^{s}\right)}^{2}\right)}\\ \mathrm{ARMSE}\overset{\Delta }{=}\sqrt{\frac{1}{MN}\sum_{k=1}^{N}\sum_{s=1}^{M}\left({\left(x_{k}^{s}-{\hat{x}}_{k}^{s}\right)}^{2}+{\left(y_{k}^{s}-{\hat{y}}_{k}^{s}\right)}^{2}\right)} \end{array}\right.$$

where $\left(x_{k}^{s},y_{k}^{s}\right)$ and $\left({\hat{x}}_{k}^{s},{\hat{y}}_{k}^{s}\right)$ are the true and estimated positions(or velocity) at the *k*th step of the *s*th Monte Carlo run, and $\mathrm{RMS}E_{pos}\left(k\right)$ is the RMSE of position at *k* step. The ARMSEs of position and velocity are denoted as $\mathrm{ARMS}E_{pos}$ and $\mathrm{ARMS}E_{vel}$, respectively [12,21].

In order to verify whether the proposed filtering algorithm improve the solution of accuracy degradation problem of original MCKF in no-stationary non-Gaussian noise conditions, the simulation was divided into two stages, and the noises with different distributions were used to test the performance of the proposed filter. To simulate the non-Gaussian noise sequence contaminated by impulsive noise interference, the Gaussian mixture distribution model with specific parameters are used to generate the noise.

The number of Monte Carlo runs was *M* = 1000 and the specific noise parameters were set as follows:

$$\begin{array}{c} \left\{\begin{array}{c} w_{k}∼0.95N\left(0,Q_{k}\right)+0.05N\left(0,100Q_{k}\right),\mathrm{stage}1:1<t\leq N/2\\ w_{k}∼0.90N\left(0,Q_{k}\right)+0.10N\left(0,100Q_{k}\right),\mathrm{stage}2:N/2<t\leq N \end{array}\right.\\ \left\{\begin{array}{c} v_{k}∼0.90N\left(0,R_{k}\right)+0.10N\left(0,100R_{k}\right),\mathrm{stage}1:1<t\leq N/2\\ v_{k}∼0.95N\left(0,R_{k}\right)+0.05N\left(0,100R_{k}\right),\mathrm{stage}2:N/2<t\leq N \end{array}\right. \end{array}$$

**Figure 1:**
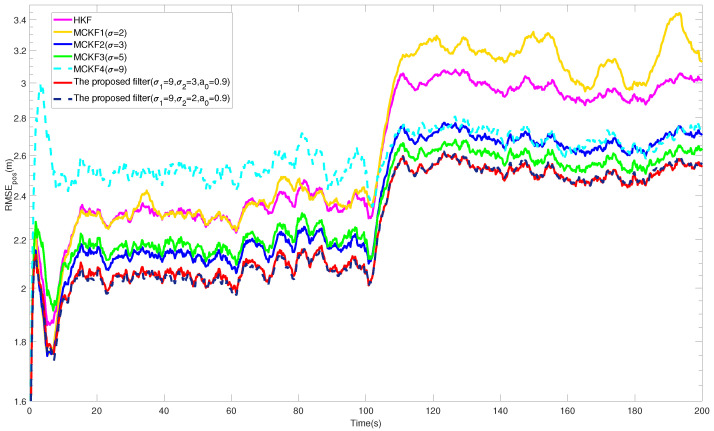
RMSEs of the position from different filters.

**Figure 2:**
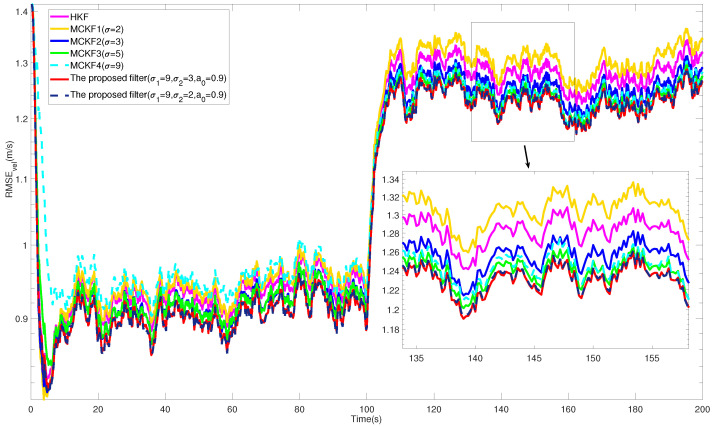
RMSEs of the velocity from different filters.

The RMSEs of the position and velocity from different filters are shown in Figure 1 and Figure 2. In addition, the ARMSEs from different filters at all stages are listed in Table 1 for comparison. In the intervals $\left(0,N/2\right]$ and $\left(N/2,N\right]$, the process and measurement vectors were contaminated by non-Gaussian heavy-tailed noise with different distributions. The plots show that the RMSEs from different filters have obvious differences, and the best kernel size for the MCKF at each stage varied accordingly. For example, MCKF2 (σ = 3) and MCKF3 (σ = 5) achieved higher accuracy, respectively, at these two stages; nevertheless, a too large or small kernel size also resulted in the filtering performance to degrade or even diverge, as shown by MCKF1 (σ = 2) and MCKF4 (σ = 9).

In comparison, the proposed filter had the lowest estimation error at each stage, especially in the case of noise distribution change, the results show that the proposed VB interference method played a positive role in the filtering process, which takes into account both stability performance and robustness to non-Gaussian noise. Therefore, by comparing the filtering performance of all stages, the superiority of the proposed algorithm is preliminarily verified.

For the existing robust filters with fixed parameters, such as the classic MCKF or HKF, the filtering parameters can be obtained by experience or trial and error methods, which are more applicable to stationary noise conditions. However, as mentioned before, the classic MCKF does not always achieve satisfactory estimation accuracy in non-stationary noise environment. In order to show this change more concretely, the process and measurement noise distribution can be expressed as $w_{k}∼\left(1-p_{1}\right)N\left(0,Q_{k}\right)+p_{1}N\left(0,100Q_{k}\right)$ and $v_{k}∼\left(1-p_{2}\right)N\left(0,R_{k}\right)+p_{2}N\left(0,100R_{k}\right)$, where $p_{1}$ and $p_{2}$ represent the outlier percentage of noise.

**Figure 3:**
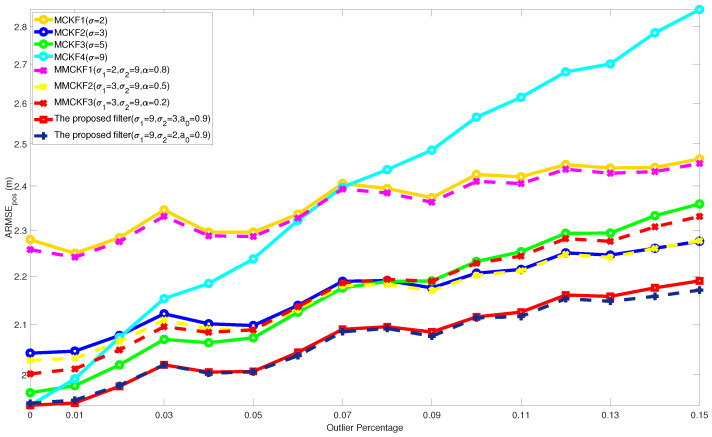
ARMSEs of position from different filters with varying $p_{2}$.

**Figure 4:**
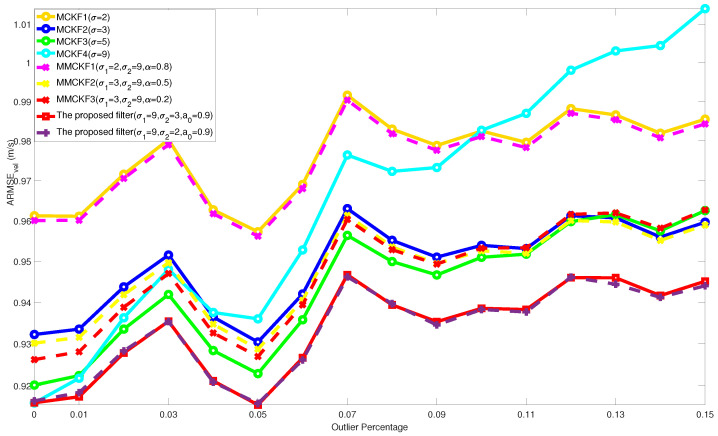
ARMSEs of velocity from different filters with varying $p_{2}$.

In Figure 3 and Figure 4, the outlier percentage of process noise was fixed as $p_{1}$ = 0.05, and the ARMSEs of the different filters varying with $p_{2}$: 0 ∼ 0.15 are shown. The overall performance of the ARMSEs showed an increasing trend, which means that as the proportion of impulsive noise increased, the estimation accuracy of the filters also decreased. In this test, the MCKFs with different kernel parameters obtained significant filtering accuracy differences. This means that, under the interference of different non-Gaussian noise, the MCKFs with a fixed kernel parameter could not ensure reliable estimation results, and the MCKFs lack enough self-adaptive ability.

In addition, the fixed-parameters MMCKF without variational Bayesian iteration also taken for comparison. The comparison shows that the results of MMCKF always converges to the MCKF of a specific fixed parameter. Therefore, although it achieves better accuracy than MCKF in some cases, it does not avoid similar accuracy degradation problem as MCKF. Since the filtering results of MMCKF are similar to the typical MCKF with specific parameters, therefore, the comparison and discussion of the simulation mainly focus on the classic MCKF.

Compared with other filtering results, it can be concluded that the proposed filter has better performance than other existing algorithms. On the one hand, the algorithm does not lose much optimality of estimation caused by changing the objective function of KF, and on the other hand, it retains robustness to the increased impulsive noise probability. Therefore, the proposed filter further demonstrates its performance advantage in various non-Gaussian noise environments.

In addition, the simulation tests were performed to compare the filtering performance with different initial parameter configurations. For the proposed IMMCKF, the Gaussian correntropy $G_{\sigma_{1}}\left(e\right)$, $G_{\sigma_{2}}\left(e\right)$ were mixed to generate the mixture correntropy, where $\sigma_{1}$ and $\sigma_{2}$ represent the specific kernel parameters, and $\sigma_{1}>\sigma_{2}$. It has been proved that Gaussian correntropy with a smaller kernel bandwidth is more sensitive to impulsive noise. However, this also degrades the filtering stability and might lead to divergence.

Table 2 lists the RMSEs of the proposed filter with different $\sigma_{1}$ and $\sigma_{2}$. In general, the filtering results of the proposed filter with different $\sigma_{1}$ and $\sigma_{2}$ were not significant. Within a certain range, the filtering accuracy improved with the increase of $\sigma_{1}$, as it provided better compatibility to Gaussian noises for the filtering algorithm. As too large of a kernel bandwidth may reduce Gaussian correntropy’s robustness to impulsive noise, $\sigma_{1}$ much greater than $\sigma_{2}$ would not be considered as the parameter set. Therefore, generally speaking, it is easy to choose appropriate $\sigma_{1}$ and $\sigma_{2}$ for the proposed algorithm.

In this case, several potential shape parameter options were used for comparison. Table 3 lists the ARMSEs of position and velocity from the proposed filter with different initial shape parameters. As demonstrated, the estimation accuracy can be improved somewhat by fine-tuning the parameter within the given range, but in general, it does not significantly affect the overall filtering performance. In summary, for the state estimation problem in non-stationary noises, the filter proposed in this work has good compatibility with the initial parameter sets.

**Figure 5:**
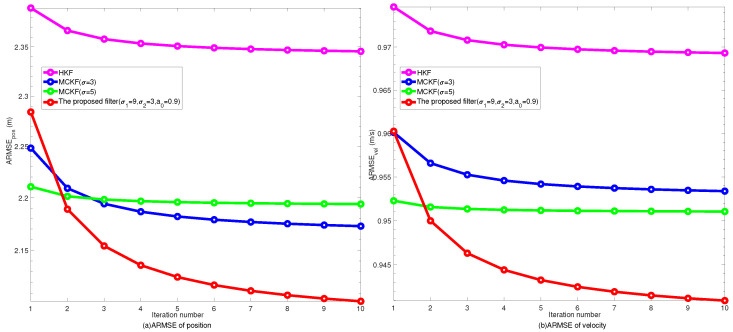
ARMSEs versus iteration number from different filters.

In order to balance the computational efficiency and the filtering performance, it is necessary to choose a reasonable number of iterations for the proposed filter. Figure 5 shows the ARMSEs of filters with different numbers of iterations. For comparison, several filtering results with similar performance in the simulation are also taken into accounts. It can be concluded that the accuracy of the proposed algorithm can be greatly improved after several iterations, and the filtering result also gradually converged. In practical applications, the increase of iteration times brings more computational burden. As shown from Figure 5, when the number of iterations $N_{m}$ is 3∼5, the filtering algorithm has obtained satisfactory estimation accuracy. Therefore, when considering the accuracy and calculation burden factors, the number of iterations $N_{m}$ set as 3∼5 is reasonable.

The implementation time of the proposed filter and existing filters in a single-step run with $N_{m}$ = 1 is evaluated. It shows that KF (0.0054 ms) is the fastest, MCKF (0.0253 ms), HKF (0.0251 ms) and MMCKF (0.0212 ms) has similar computational burden and the proposed IMMCKF (0.0490 ms). Compared with the MCKF and MMCKF, the computation complexity of the proposed filter increased simultaneously due to the additional variational Bayesian iterations. In view of this, the additional computation cost of the IMMCKF can be compensated by adjusting the number of iterations. Therefore, it is still feasible to apply this algorithm in real-time applications.

### 4.2. Example II: INS/GPS Integrated Navigation System

**Figure 6:**
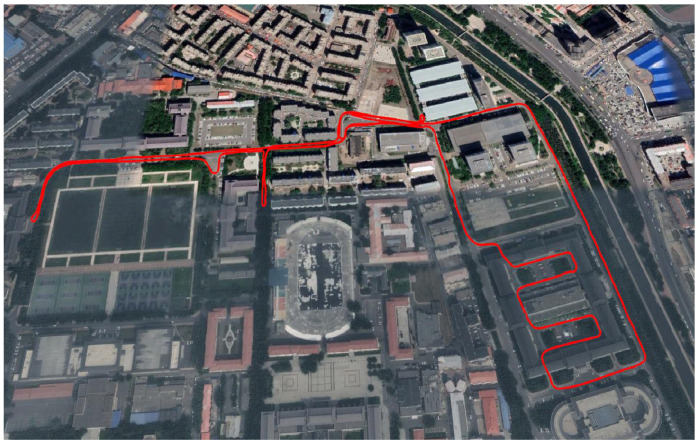
The test trajectory of the vehicle.

To validate the effectiveness and superiority of the proposed algorithm in this paper, the experimental data collected in a vehicle-mounted INS/GPS integrated navigation experiment was used for the test. The experiment was carried out in the campus of Harbin Engineering University, the test trajectory is shown in Figure 6. A low-cost MEMS-IMU based INS/GPS integrated navigation system is used to provide the navigation data.

An INS/GNSS integration navigation system includes a self-made navigation-grade fiber optic strap-down INS and a double-antenna GPS receiver is used for reference. The initial velocity and position of INS/GPS are obtained directly from GPS measurement, the initial level attitude information is acquired from the alignment results of high accuracy SINS. In the experimental test, the car moves along a bumpy road, and the GPS might work abnormally due to the occlusion of trees and buildings.

The sampling frequencies of the low-cost IMU and GPS were 100 Hz and 1 Hz, respectively. The loosely coupled configuration is used in the integration, and a linear closed-loop feedback scheme as [15,23] is used. The state variables vector is defined as $x_{k}={\left[\varphi^{n}\;\delta v^{n}\;\delta p^{n}\;\varepsilon^{b}\;\nabla^{b}\right]}^{T}$, where $\varphi^{n}$, $\delta v^{n}$, $\delta p^{n}$ denote the attitude error, velocity error and position error expressed in the n-frame, respectively. The bias of the gyro and accelerometer are $\varepsilon^{b}=10^{{}^{\circ }}/h$ and $\nabla^{b}$ = 100 μg, respectively. The initial estimation was ${\hat{x}}_{0|0}=0_{15\times 1}$. The process noise vector is expressed as $w={\left[w_{gx}^{b}\;w_{gy}^{b}\;w_{gz}^{b}\;w_{ax}^{b}\;w_{ay}^{b}\;w_{az}^{b}\right]}^{T}$, where $w_{g}^{b}=1^{\circ }/\sqrt{h}$ and $w_{a}^{b}=1500\;\mu g/\sqrt{\mathrm{Hz}}$, respectively, and the measurement noise covariance matrix $R\;=\;\mathrm{diag}(0.5\;m/s\cdot I_{3}\;\;1.5\;m\cdot I_{3})$. The initial position and velocity errors were set as $\delta p={\left[1.5\;m\;1.5\;m\;1.5\;m\right]}^{T}$, $\delta V^{n}={\left[0.1\;m/s\;0.1\;m/s\;0.1\;m/s\right]}^{T}$, and attitude errors $\phi \;=\;{\left[0.1^{{}^{\circ }}\;0.1^{{}^{\circ }}\;0.1^{{}^{\circ }}\right]}^{T}$.

The filter only performs time update while there are no GPS outputs. To compare the robustness of different filters to non-Gaussian measurement noise interference, inspired by the scheme in [34,35], the gross errors were added artificially to the measurement data. In the integrated navigation system, the velocity and position state variables are susceptible to external interference. Therefore, the velocity and position errors are used for comparison.

**Figure 7:**
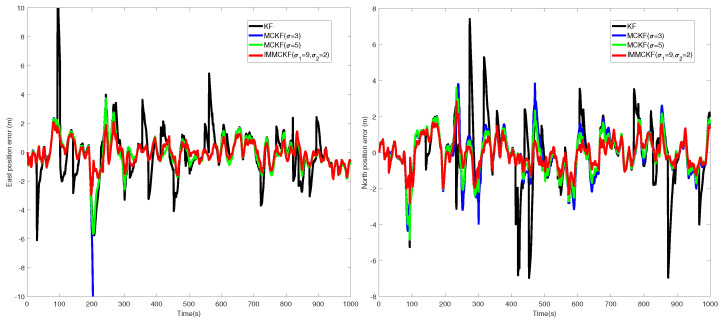
The position errors from different filters in non-Gaussian noises.

**Figure 8:**
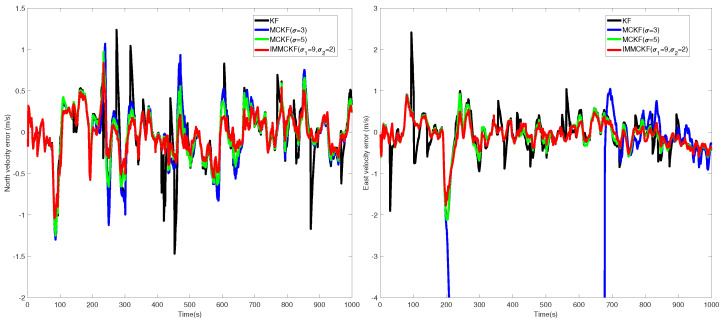
The velocity errors from different filters in non-Gaussian noises.

For comparison, several typical filtering results are shown in Figure 7 and Figure 8. It can be seen that most filters obtain similar results in stable periods, as the measurement output is reliable in these periods. For the existing robust filters with fixed-parameter, due to the uncertain interference factors in practical application. In this example, both MCKF1 (σ = 2) and MCKF2 (σ = 3) have a divergence trend in the filtering process. In contrast, MCKFs with larger kernel parameters and the proposed filter have more stable performance.

In this example, the measurement sequence is disturbed by impulsive noise with a certain probability. It is evident from the Figures that the estimation accuracy of KF is seriously corrupted during filtering. Generally, a small kernel size can be more effective in attenuating the measurement outliers, but at the same time, it inevitably decreases the filter’s stability, e.g., MCKF1 (σ = 2) failed to obtain reliable filtering results due to divergence. By comparing the robustness of MCKF3 (σ = 5), MCKF4 (σ = 9), and other algorithms to non-Gaussian noise, it can be concluded that, for existing MCKFs, it is difficult to obtain both stable and robustness estimation results.

The results listed in Table 4 confirmed that the filter proposed in this work solves the problem well. NaN represents invalid output due to filter divergence. Compared with other robust filters, the proposed filter achieves better filtering results by taking into account both the robustness and stability of the filter, which demonstrated the conclusions of the previous simulation example.

## 5. Conclusions

In this work, the performance degradation problem of the existing MCKF in non-stationary noises is explored, and a new improved mixture correntropy filtering algorithm is proposed as an effective solution. To cope with the dynamic process where both Gaussian and non-Gaussian noises may occur, the intermediate random variables is used to construct the mixture correntropy. By derivation, the state variables and intermediate parameters are approximated via a variational Bayesian approach. The theoretical derivation and numerical test results show that the proposed method significantly improves upon the existing MCKF algorithms in different conditions, which offers a promising improvement for the robust filtering problem in complex noise environments.

## Figures and Tables

**Table 1:** ARMSEs of different filters at each stage.

Filters	Stage 1		Stage 2		All Stages	
	Position (m)	Velocity (m/s)	Position (m)	Velocity (m/s)	Position (m)	Velocity (m/s)
KF	4.014	1.207	3.420	1.302	3.729	1.255
HKF	2.347	0.970	2.945	1.268	2.663	1.129
MCKF1 (σ=2)	2.346	0.977	3.081	1.290	2.738	1.144
MCKF2 (σ=3)	2.167	0.953	2.659	1.242	2.426	1.107
MCKF3 (σ=5)	2.201	0.953	2.572	1.226	2.394	1.098
MCKF4 (σ=9)	2.552	0.993	2.683	1.231	2.618	1.118
IMMCKF1 (σ1=9,σ2=3)	2.084	0.939	2.503	1.218	2.303	1.087
IMMCKF2 (σ1=9,σ2=2)	2.078	0.939	2.508	1.218	2.303	1.088

**Table 2:** ARMSEs of the proposed filter with different $\sigma_{1}$ and $\sigma_{2}$.

Filters	Stage 1		Stage 2		All Stages	
	Position (m)	Velocity (m/s)	Position (m)	Velocity (m/s)	Position (m)	Velocity (m/s)
KF	4.014	1.207	3.420	1.302	3.729	1.255
IMMCKF1 ($\sigma_{1}=6,\sigma_{2}=3$)	2.117	0.943	2.530	1.221	2.332	1.091
IMMCKF2 ($\sigma_{1}=6,\sigma_{2}=2$)	2.108	0.942	2.532	1.222	2.330	1.091
IMMCKF3 ($\sigma_{1}=9,\sigma_{2}=3$)	2.084	0.939	2.503	1.218	2.303	1.087
IMMCKF4 ($\sigma_{1}=9,\sigma_{2}=2$)	2.078	0.939	2.508	1.218	2.303	1.088
IMMCKF5 ($\sigma_{1}=12,\sigma_{2}=3$)	2.074	0.938	2.494	1.217	2.294	1.086
IMMCKF6 ($\sigma_{1}=12,\sigma_{2}=2$)	2.054	0.936	2.502	1.218	2.289	1.086

**Table 3:** ARMSEs of the proposed filter with different $a_{0}$.

Filters	Stage 1		Stage 2		All Stages	
	Position(m)	Velocity (m/s)	Position (m)	Velocity (m/s)	Position (m)	Velocity (m/s)
KF	4.014	1.207	3.420	1.302	3.729	1.255
IMMCKF1 ($a_{0}$ = 0.94)	2.118	0.943	2.514	1.218	2.324	1.089
IMMCKF2 ($a_{0}$ = 0.92)	2.095	0.940	2.505	1.218	2.309	1.088
IMMCKF3 ($a_{0}$ = 0.90)	2.084	0.939	2.503	1.218	2.303	1.087
IMMCKF4 ($a_{0}$ = 0.88)	2.079	0.938	2.504	1.218	2.301	1.087
IMMCKF5 ($a_{0}$ = 0.86)	2.077	0.938	2.510	1.219	2.304	1.088

**Table 4:** The RMSEs of position, velocity from different filters.

Filtering Algorithms	PosE (m)	PosN (m)	PosU (m)	VelE (m/s)	VelN (m/s)	VelU (m/s)
KF	1.736	1.726	0.799	0.466	0.363	0.118
HKF	1.817	1.428	0.681	0.488	0.320	0.112
MCKF1 (σ = 2)	NaN	NaN	0.675	NaN	NaN	0.117
MCKF2 (σ = 3)	NaN	1.187	0.671	NaN	0.333	0.112
MCKF3 (σ = 5)	1.069	1.061	0.671	0.379	0.290	0.111
MCKF4 (σ = 9)	1.121	1.111	0.674	0.385	0.300	0.111
MMCKF1 ($\sigma_{1}$ = 2, $\sigma_{2}$ = 9, α = 0.8)	NaN	NaN	0.711	NaN	NaN	0.120
MMCKF2 ($\sigma_{1}$ = 3, $\sigma_{2}$ = 9, α = 0.5)	1.221	0.995	0.674	0.400	0.275	0.112
MMCKF3 ($\sigma_{1}$ = 3, $\sigma_{2}$ = 9, α = 0.2)	1.026	1.007	0.673	0.374	0.278	0.111
IMMCKF1 ($\sigma_{1}$ = 9, $\sigma_{2}$ = 3)	0.888	0.903	0.657	0.345	0.261	0.110
IMMCKF2 ($\sigma_{1}$ = 9, $\sigma_{2}$ = 2)	0.751	0.775	0.640	0.319	0.238	0.110

## Data Availability

No new data were created or analyzed in this study. Data sharing is not applicable to this article.
